# Clinical Statistics of Dysphagia Rehabilitation Provided on Dental Visits to a Partner Hospital

**DOI:** 10.1155/2022/5952423

**Published:** 2022-04-11

**Authors:** Mai Ohkubo, Atsushi Hanzawa, Keina Miura, Tetsuya Sugiyama, Ryo Ishida, Ken-ichi Fukuda

**Affiliations:** ^1^Department of Oral Health and Clinical Science, Division of Dysphagia Rehabilitation, Tokyo Dental College, Chiyoda-ku, Tokyo 101-0061, Japan; ^2^Department of Oral Health and Clinical Science, Division of Special Needs Dentistry and Orofacial Pain, Tokyo Dental College, Chiyoda-ku, Tokyo 101-0061, Japan; ^3^Tokyo Dental College Chiba Dental Center, Division of Dysphagia Rehabilitation, Division of General Dentistry, Chiba 261-8502, Japan

## Abstract

Maintaining oral hygiene is an important yet often neglected aspect of rehabilitation medicine. Our visiting dental team, which provides dental treatments and swallowing rehabilitation, partnered with a medical hospital that had no dental department and began visiting and treating inpatients at this hospital. This study is aimed at evaluating the effects of dysphagia rehabilitation, and this was jointly conducted by medical and dental hospitals. The survey was conducted between May 2017 and March 2018. We retrospectively examined dysphagia rehabilitation provided to 25 patients (12 men and 13 women) aged 40–92 years (mean age: 77.1 ± 12.3 years). The largest number of requests for dental treatment was received from the internal medicine department (13 requests, 52.0%). A total of 39 videofluoroscopic or videoendoscopic examinations of swallowing interventions for dysphagia rehabilitation were conducted. All patients' oral and swallowing functions were evaluated using the functional oral intake scale (FOIS). At initial assessment, 9, 13, and 0 patients were at FOIS levels 1, 2, and 3 (use of tube feeding), respectively, and 1, 2, and 0 patients were at FOIS levels 4, 5, and 6 (only oral feeding), respectively. At the final assessment, 6, 10, and 4 patients were at FOIS levels 1, 2, and 3, respectively, and 0, 2, and 3 patients were at FOIS levels 4, 5, and 6, respectively. Oral and swallowing functions differed significantly between the first and final visits (*p* = 0.02). Visits conducted by a team of oral health practitioners to a medical hospital without a dental department appear to have a major impact and will become even more important in the future.

## 1. Introduction

There are concerns about the decline in oral function [1] and poor oral hygiene [2] among the elderly. Dental treatment and oral hygiene management provided through a collaboration between the medical and dental departments are important in improving the quality of life (QOL) of older adults being treated for numerous systemic diseases. Moreover, the relationship between oral and systemic health has been widely reported, including the efficacy of oral hygiene management in preventing aspiration pneumonia [3].

According to a previous report, oral hygiene management significantly improved oropharyngeal dysphagia within a short period [4]. In addition, early and systematic screening for dysphagia and enhanced oral hygiene reduced the incidence of pneumonia [5].

However, maintaining oral hygiene is an important yet often neglected aspect of rehabilitation medicine. Poor oral hygiene and oral function directly impact dental occlusion and chewing ability. Furthermore, malnutrition and dysphagia have a direct impact on functional recovery [6]. Approximately 71% of hospitalized rehabilitation patients and 91% of hospitalized acute care patients have compromised oral health [6]. Moreover, as of 2017, only 14.9% of hospitals in Japan had dental care-related departments [7], making medical-dental partnerships an urgent requirement. In this study, the impact of dysphagia rehabilitation on the oral intake status of inpatients was evaluated. This study is aimed at descriptively examining and comparing the effects of swallowing function between the first and last dental examinations according to the level of the functional oral intake scale (FOIS).

## 2. Materials and Methods

### 2.1. Participants

**Figure 1:**
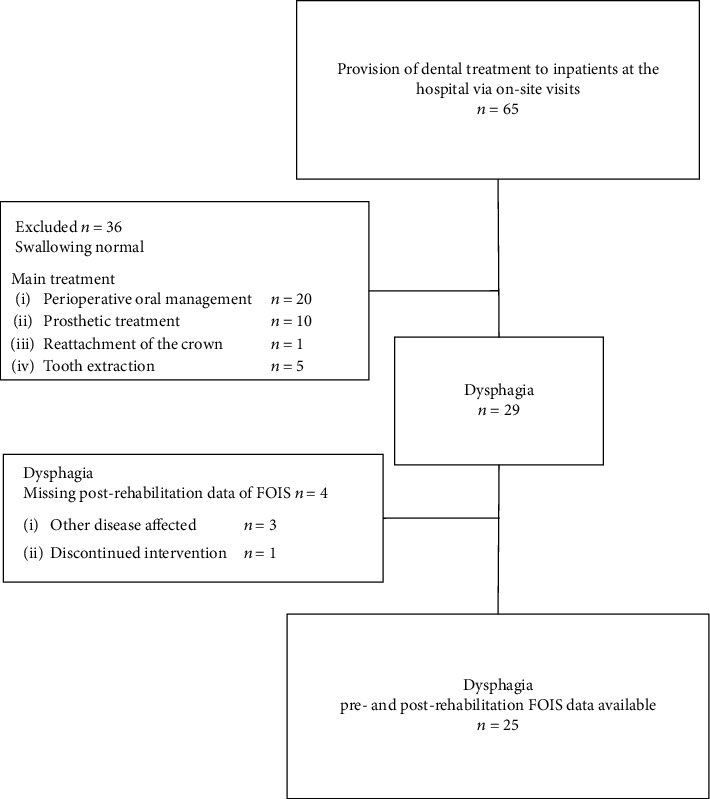
Participant flowchart.

This study was approved by the Ethical Review Board of Tokyo Dental College (approval no. 864). The survey was conducted between May 2017 and March 2018. We enrolled 65 patients (26 men and 39 women) aged 40–101 years who were treated by our dental team at a hospital without a dental department. Of these, 36 did not require dysphagia rehabilitation but received only oral hygiene management. Moreover, of the 29 patients with dysphagia, four could not be assessed for FOIS before and after treatment (Figure 1). The sample size was calculated using G∗Power 3.1.9.7. With *α* set at 0.05, 25 participants would be required for the pilot study to achieve a power of 0.80 to detect a 20% difference in mean knowledge scores. Our dental team visited the medical hospital twice per week. The hospital had 198 beds in total (138 beds in the acute general unit, 40 in the community comprehensive care unit, and 20 in the palliative care unit).

### 2.2. Methods

This study is a descriptive and longitudinal study. Patient characteristics, including age, sex, and underlying disease, were examined in the departments requesting care. Dysphagia rehabilitation was requested by the patients and their families or by attending physicians, nurses, and speech therapists who identified patients in need of such care, such as those with oral discomfort and aspiration pneumonia due to poor swallowing function. Dysphagia rehabilitation was requested and provided after the patients, and their families received an adequate explanation and provided informed consent.

First, patients who needed oral hygiene or had poor swallowing conditions were referred for dental care. Early dental treatment is important for maintaining and improving oral function and oral hygiene and can help achieve a proper swallowing function.

Thereafter, dysphagia screening assessments were conducted by a speech therapist. In cases of abnormal swallowing, the visiting dentist performed videofluoroscopic and/or videoendoscopic examinations of swallowing interventions. Videofluoroscopy comprises X-ray imaging with a contrast agent using a fluoroscope. It helps observe in real time the flow of food from the mouth to the pharynx and esophagus associated with the swallowing motion. Videoendoscopy is a direct fiberscope examination of the closure of the glottis and whether saliva and secretions remain in the pharynx [8]. The dysphagia rehabilitation team comprising speech therapists, nurses, dental hygienists, and dentists was involved in the dysphagia rehabilitation program. A speech therapist and nurse performed daily dysphagia rehabilitation. The dentists evaluated swallowing function and the progress of the dysphagia rehabilitation program once a week. Inpatients underwent professional tooth cleaning and scaling to remove dental plaque and calculus. Oral hygiene management was performed by a dental hygienist once a week. Patients who received therapy for oral and swallowing functions were evaluated using the FOIS at the initial examination and after therapy. The FOIS grades ranging from levels 1 to 7 were determined for each patient, with levels ≤ 3 indicating tube feeding (Table 1) [9]. Swallowing functions were compared between the first and final dental examinations based on the FOIS.

### 2.3. Statistical Analysis

Statistical analysis was performed using SPSS Statistics 23.0 (SPSS Japan Inc., Tokyo, Japan). The Wilcoxon signed-rank test was used to compare the FOIS levels among patients.

## 3. Results

We retrospectively examined the dysphagia rehabilitation provided to 25 patients (12 men and 13 women) aged 40–92 years (mean age: 77.1 ± 12.3 years). The mean age of the men and women in the patient cohort was 74.9 ± 13.4 and 79.4 ± 10.6 years, respectively, and were not significantly different (*p* = 0.38). The largest number of requests for dental treatment was received from the internal medicine department (13 requests, 52.0%; Table 2). The most common underlying disease was malignant neoplasm (10 patients, 27.0%), followed by pneumonia (8 patients, 21.6%; Table 3).

A total of 91 (58.3%) oral hygiene interventions were performed, followed by 39 videofluoroscopic or videoendoscopic examinations of swallowing interventions for dysphagia rehabilitation (Table 4).

**Figure 2:**
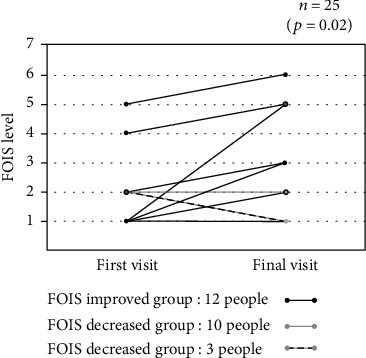
Distribution of changes in FOIS levels. FOIS: functional oral intake scale.

Oral and swallowing functions were evaluated in all patients using the FOIS.  Table 5 shows the results of the initial and final assessments. The oral and swallowing functions differed significantly between the first and final visits (*p* = 0.02; Figure 2). The FOIS level increased in 12 patients (improved group), remained unchanged in 10 (unchanged group), and decreased in three (decreased group).

## 4. Discussion

Oral hygiene management is recommended to prevent systemic infections caused by oral pathogens [10, 11]. Interest in oral hygiene management is growing because it has been reported to prevent aspiration pneumonia, improve nutritional status, and promote rehabilitation. Oral hygiene management has been demonstrated to reduce the risk of ventilator-associated pneumonia and postoperative complications, such as hospital-acquired pneumonia, in patients with good oral hygiene [12–14]. The large number of dental treatment requests from the oncology department for patients with malignant neoplasms was probably because changes in the oral environment caused by chemotherapy required oral hygiene management, which created a demand for dental hygienists. In recent years, swallowing function therapy before, during, and after chemoradiotherapy has improved function and QOL parameters [15]. Furthermore, interventions by dental hygienists enabled patients to continue oral hygiene management based on specialized assessments and comprehensive instructions from dentists, resulting in a higher quality of specialized oral hygiene management. Moreover, efforts to improve QOL should include early dental interventions to prevent infections and postoperative complications during hospitalization. Oral hygiene management provided by dental hygienists improves not only oral status, swallowing function, and nutritional status but also activities of daily living, home discharge, and in-hospital mortality in postacute rehabilitation [16].

Dysphagia rehabilitation accounts for 13% of dental interventions, indicating a demand for assessing oral and swallowing functions and dysphagia rehabilitation during hospitalization. Of the 25 patients assessed in the present study, 12 were finally classified as improved and 10 as unchanged. Thus, assessments of oral and swallowing function, consultations with speech therapists and other medical personnel, and the development of dysphagia rehabilitation programs were all affected.

In addition, interventions provided in the hospital may have enabled dysphagia rehabilitation at an early stage. It is reported that early dysphagia rehabilitation is associated with shorter hospital stays and fewer days between hospitalization and oral intake [17]. Our results also demonstrated improvement in swallowing function and FOIS values in many cases. Therefore, implementing early oral hygiene management by dentists, dental hygienists, and medical-dental cooperation in rehabilitation is necessary.

In a previous study, dental referrals by medical professionals were frequent in 32% of the cases and infrequent in 68% [6]. Furthermore, although dentists were interested in expanding the medical-dental collaboration, many general practitioners did not see the need for the collaboration [18].

Considering these factors, it is even more important to strengthen medical partnerships, which may be achieved by establishing interhospital systems that include clerical and other staff, allowing hospitals without dental departments to share highly specialized patient information efficiently.

This study had some limitations. First, the patient background was heterogeneous, and the number of patients was small (25). Furthermore, since the participants were patients with dysphagia, a control group without swallowing rehearsal could not be established for ethical reasons. Therefore, this study could not control for confounding factors that may have contributed to improving swallowing function. However, we believe that the effects of oral hygiene management, diagnosis of swallowing function, and consultation with the visiting speech-language pathologist to plan and conduct swallowing rehabilitation were enhanced.

Moreover, visiting the hospital to provide dental treatment enabled practitioners to meet patients face-to-face, and practitioners of different specialties could be involved in the process. This type of cross-disciplinary cooperation may facilitate future oral health consultations. It is important to investigate teamwork involving dental hygienists and areas related to the efficiency of dental treatment in the future.

## 5. Conclusions

Oral and swallowing functions differed significantly between the first and final assessments. Visits conducted by a team of oral health practitioners in a medical hospital without a dental department appear to have a significant impact on oral and swallowing functions. We must strive to improve the patients' QOL by providing safer and higher-quality dysphagia rehabilitation and oral hygiene by pursuing partnerships between dental hospitals and medical hospitals without dental departments in the future.

## Figures and Tables

**Table 1:** FOIS (functional oral intake scale).

Level 1: nothing by mouth.
Level 2: tube-dependent with minimal attempts of food or liquid.
Level 3: tube-dependent with consistent oral intake of food or liquid.
Level 4: total oral diet of a single consistency.
Level 5: total oral diet with multiple consistencies but requiring special preparation or compensations.
Level 6: total oral diet with multiple consistencies without special preparation, but with specific food limitations.
Level 7: total oral diet with no restrictions.

**Table 2:** Number of patients requesting dental care by department.

Requesting department	Patients (*n* (%))
Internal medicine	13 (52.0)
Gastrointestinal surgery	5 (20.0)
Oncology	4 (16.0)
Gastrointestinal and liver treatment	3 (12.0)
Total	25 (100)

**Table 3:** Number of patients with underlying conditions (some patients had multiple underlying conditions).

Condition	Patients (*n* (%))
Malignant neoplasm	10 (27.0)
Pneumonia	8 (21.6)
Stroke	5 (13.5)
Dementia	3 (8.1)
Parkinson's disease	3 (8.1)
Other	8 (8.1)

**Table 4:** Number of cases by type of dental treatment (some patients had multiple underlying treatments).

Content	Cases (%)
Oral hygiene management	91 (58.3)
Videofluoroscopic and/or videoendoscopic examinations	39 (25.0)
Eating/swallowing function therapy	25 (16.0)
Prosthetic treatment	1 (0.6)
Total	156 (100.0)

**Table 5:** Changes in the FOIS level for eating and swallowing functions between the initial and final assessments.

	Number of patients (*n* (%))	
FOIS level	Initial assessment	Final assessment
Level 1	9 (36.0)	6 (24.0)
Level 2	13 (52.0)	10 (40.0)
Level 3	0 (0)	4 (16.0)
Level 4	1 (4.0)	0 (0.0)
Level 5	2 (8.0)	3 (12.0)
Level 6	0 (0.0)	2 (8.0)
Level 7	0 (0.0)	0 (0)
Total	25 (100.0)	25 (100.0)

## Data Availability

The data used to support the findings of this study are included in this article. Data are also available from the corresponding author upon request.
